# The potential for deprescribing in care home residents with Type 2 diabetes

**DOI:** 10.1007/s11096-016-0323-4

**Published:** 2016-05-30

**Authors:** Lillan Mo Andreassen, Reidun Lisbet Skeide Kjome, Una Ørvim Sølvik, Julie Houghton, James Antony Desborough

**Affiliations:** Department of Global Public Health and Primary Care, University of Bergen, Kalfarveien 31, PO Box 7804, 5020 Bergen, Norway; School of Health Sciences, University of East Anglia, Norwich Research Park, Norwich, NR4 7TJ UK; School of Pharmacy, University of East Anglia, Norwich Research Park, Norwich, NR4 7TJ UK

**Keywords:** Care homes, Deprescribing, Medicines optimisation tool, Pharmacists, Potentially inappropriate medicines, Type 2 diabetes mellitus

## Abstract

*Background* Type 2 diabetes is a common diagnosis in care home residents that is associated with potentially inappropriate prescribing and thus risk of additional suffering. Previous studies found that diabetes medicines can be safely withdrawn in care home residents, encouraging further investigation of the potential for deprescribing amongst these patients. *Objectives* Describe comorbidities and medicine use in care home residents with Type 2 diabetes; identify number of potentially inappropriate medicines prescribed for these residents using a medicines optimisation tool; assess clinical applicability of the tool. *Setting* Thirty care homes for older people, East Anglia, UK. *Method* Data on diagnoses and medicines were extracted from medical records of 826 residents. Potentially inappropriate medicines were identified using the tool ‘Optimising Safe and Appropriate Medicines Use’. Twenty percent of results were validated by a care home physician. *Main outcome measure* Number of potentially inappropriate medicines. *Results* The 106 residents with Type 2 diabetes had more comorbidities and prescriptions than those without. Over 90 % of residents with Type 2 diabetes had at least one potentially inappropriate medication. The most common was absence of valid indication. The physician unreservedly endorsed 39 % of the suggested deprescribing, and would consider discontinuing all but one of the remaining medicines following access to additional information. *Conclusion* UK care home residents with Type 2 diabetes had an increased burden of comorbidities and prescriptions. The majority of these patients were prescribed potentially inappropriate medicines. Validation by a care home physician supported the clinical applicability of the medicines optimisation tool.

**Electronic supplementary material:**

The online version of this article (doi:10.1007/s11096-016-0323-4) contains supplementary material, which is available to authorized users.

## Impacts of practice

- The results from this study suggest that care home residents with Type 2 diabetes have a higher burden of comorbidities and polypharmacy than residents without diabetes, thereby having increased risk for potentially inappropriate prescribing.
- The evidence-based, pragmatic medicines optimisation tool used in this study allows pharmacists to identify medicines eligible for deprescribing for care home residents with Type 2 diabetes, thus reducing polypharmacy and potentially adverse events following from it.

## Introduction

In the UK, care homes for older people provide accommodation and nursing or personal care to those who need it. These institutions are staffed 24 h a day, with or without qualified nursing staff, and are referred to as nursing homes and residential homes respectively. Care home residents generally have a limited life expectancy [1] and experience high levels of disability, comorbidity and polypharmacy [2]. Non-insulin-dependent diabetes, also known as Type 2 diabetes mellitus (T2DM), is reported to be among the ten most common diagnoses, affecting 15 % of the care home population [2].

T2DM is associated with a range of comorbidities and complications [3, 4], deteriorating health and reducing quality of life. In the general older population, diabetes has been identified as a predictor of multiple medicine use [5] and an independent risk factor for being prescribed potentially inappropriate medicines or combinations of these [6, 7]. Unnecessary or inappropriate medicines can cause adverse events and additional suffering in this already vulnerable group of patients. It is argued that people with diabetes who suffer from multiple comorbidities, cognitive impairment or reside in a long-term nursing facility may experience limited or uncertain benefit from diabetes treatment [8, 9]. Concerns about overtreatment with blood glucose lowering medicines have been reported [10, 11] and a Swedish study suggests that diabetes medicines can be safely reduced or withdrawn in the majority of these residents [11]. These findings indicate that the potential for deprescribing should be investigated to a greater extent in this population.

Deprescribing is defined by Reeve et al. [12] as «the process of withdrawal of an inappropriate medication, supervised by a health care professional with the goal of managing polypharmacy and improving outcomes». Deprescribing is increasingly acknowledged as an important part of prescribing when managing patients with multiple conditions and limited life expectancy [13–15]. Several tools exist to help determine medication appropriateness in older persons, the STOPP/START criteria [16] perhaps being the most commonly used in UK settings. However, it has been argued that whilst these criteria are useful in aiding prescribing for healthier older persons, they may be less suitable for use in settings where the patients are frail, late in life, and suffer from multiple illnesses [13]. Hence, there is a requirement for clearer practical guidance that directly addresses appropriate removal of medicines in these patients [13], that should be founded on questions about whether the medicine is currently indicated, safe and beneficial considering comorbidities [17, 18]. The NHS PrescQIPP document ‘Optimising Safe and Appropriate Medicine Use’ (OSAMU), a pragmatic, evidence-based tool, developed to allow for appropriately stopping or continuing medicines in end of life, uses such an approach [19]. When used as a resource in a care home setting, it has been shown to safely contribute to a reduction in polypharmacy, inappropriate medicines and potential adverse effects [20, 21]. In addition it contributed to a reduction in administration time, waste and costs of medicines.

## Aim of the study

This study aimed to investigate the potential for deprescribing in UK care home residents with T2DM. The objectives set were (1) to describe the comorbidities and medicine use in the residents with T2DM; (2) to describe the number of potentially inappropriate medicines in these residents using an evidence-based, pragmatic medicines optimisation tool; and (3) to describe the clinical applicability of the medicines optimisation tool used.

This study is a retrospective sub-analysis of data from the CAREMED study, a cluster randomised controlled trial investigating the impact of a multi-professional medication review service (MMRS) within 30 care homes for older people across East Anglia, UK between March 2011 and March 2013 [22].

Details of inclusion and exclusion criteria, outcome measures, data collection and ethical approval have been described in a previous publication. Findings from the main study have yet to be published.

## Ethics approval

The CAREMED study was approved by the National Health Service (NHS) Norfolk Research Ethics Committee (REC reference 09/H0310/96).

## Methods

### Data extraction and analysis

CAREMED baseline data was extracted for all 826 residents living in the 30 care homes. Data included information about the residents’ current medicines and active medical problems, derived from their medical records at the general practitioner’s (GP’s) surgery.

### Demographics

Diabetes prevalence was determined by evidence of T2DM documented as an active medical problem. Residents with other types of diabetes were excluded from the study population and further analysis. Comorbidity burden was determined from the resident’s number of active medical problems. All active medical problems in the dataset were classified according to the 22 chapters of the International Statistical Classification of Diseases and Related Health Problems 10th Revision (ICD-10) Version: 2010 [23]. Number of prescriptions was determined from the number of unique medicines prescribed. Polypharmacy was defined as prescription of ≥5 unique medicines. All medicines were coded according to the Anatomical Therapeutic Chemical (ATC) classification system [24].

### Potential for deprescribing

The NHS PrescQIPP document OSAMU consists of 46 areas for medicine optimisation based on the drug classes in the British National Formulary (BNF) chapters [19]. Based on the available CAREMED data, we identified that 35 of these areas were applicable to our population. For counting purposes, one or several explicit criteria were identified for each area by LMA in agreement with RLSK (Online Resource 1). LMA and RLSK are pharmacists with experience of clinical work and research in both community pharmacies and care homes, with particular focus on diabetes. Potentially inappropriate medicines (PIMs) were identified by LMA based on the criteria derived from the recommendations given in the OSAMU document (Online Resource 2).

As a further validation of clinical applicability of the OSAMU document a physician (CG) with clinical background from care homes, currently in involved in a large multicentre-study on medicines optimisation in care homes [25], assessed the identified PIMs for discontinuation for a random sample of 20 % of the residents. Based on the information available, the physician evaluated whether (1) the medicine could be discontinued without further question; (2) the medicine should potentially be discontinued, but not before checking other parameters of importance, e.g. laboratory values; (3) the medicine should be changed to a more appropriate choice; or (4) the medicine should be continued.

### Statistical analysis

Descriptive statistics were applied. Continuous variables are presented as medians with range and/or 95 % confidence intervals (CI), and categorical variables are presented as frequencies with percentages and/or 95 % CI. The 95 % CI for the medians and percentages were estimated by the 2.5 and 97.5 percentiles from a simple bootstrap (10.000 datasets were randomly generated for each CI). Non-overlapping CI was interpreted as significant effects. The RAND function in Microsoft Excel 2010 (Microsoft, Redmond, WA, USA) was used to create the random 20 % sample for validation. IBM SPSS Statistics 22.0 (IBM, Armonk, NY, USA) was used for all statistical analysis, apart from bootstrapping, which was performed using Python 2.7.

## Results

### Demographics, therapy and comorbidity burden

Of 826 residents, 109 had a registered diagnosis of DM. Two residents with Type 1 DM and one resident with steroid-induced diabetes were excluded, resulting in a total study population of 823 residents, where 106 residents had diagnosed T2DM (13 %). Table 1 compares residents with T2DM to residents without DM. Residents with T2DM were significantly younger and had a higher burden of both comorbidities and prescriptions than residents without DM.

**Table 1:** Demographics, burden of comorbidities and prescriptions in care home residents with and without diabetes mellitus

	Type 2 DM			No DM		
	n = 106			n = 717		
	Median	Range	[95 % CI]^a^	Median	Range	[95 % CI]^a^
Age, years	86	56–98	[84.5, 87.5]	88	39–104	[88.0, 89.0]
Age at admission, years	84	54–98	[81.0, 85.0]	86	36–103	[85.0, 86.0]
Number of active medical problems	6.5	2–16	[6.0, 7.0]	5	1–14	[4.0, 5.0]
Number of prescriptions	9	1–20	[8.5, 10.0]	7	0–27	[7.0, 7.0]

	n	%	[95 % CI]^b^	n	%	[95 % CI]^b^
Polypharmacy^c^	98	92.5	[86.7, 96.9]	534	74.5	[70.7, 78.1]
Nursing home residents	24	22.6	[8.3, 41.7]	170	23.7	[17.6, 30.0]
Women	70	66.0	[54.3, 77.1]	555	77.4	[73.9, 80.9]

*DM* diabetes mellitus

^a^Confidence intervals for median values. Non-overlapping confidence intervals are interpreted as statistically significant differences

^b^Confidence intervals for percentages. Non-overlapping confidence intervals are interpreted as statistically significant differences

^c^Polypharmacy is defined as prescription of ≥5 unique drug substances

The top five ICD-10 classifications for residents with T2DM, excluding diabetes, were I00-I99: circulatory diseases (n = 82, 77.4 %), F00-F99: mental and behavioural disorders (n = 52, 49.1 %), M00-M99: musculoskeletal and connective tissue diseases (n = 43, 40.6 %), H00-H59: eye diseases (n = 40, 37.7 %), and N00-N99: genitourinary diseases (n = 37, 34.9 %). They were treated with the following blood glucose lowering therapy: insulin only (n = 10), insulin and oral antidiabetic drugs (OADs) (n = 4), OADs only (n = 56), and no blood glucose lowering drugs (n = 36). The other most commonly prescribed groups of medicines among these residents are listed in Table 2.

**Table 2:** The most frequently prescribed drug groups in care home residents with Type 2 diabetes mellitus (n = 106)

ATC code	Therapeutic group/substance	Residents receiving therapy	
		N	%
A10	Drugs used in diabetes	70	66.0
A10A	Insulins and analogues	14	13.2
A10B	Blood glucose lowering drugs, excl. insulins	60	56.6
A10BA02	Metformin	45	42.5
A10BB09	Gliclazide	26	24.5
N02	Analgesics	65	61.3
C10	Lipid modifying agents	61	57.5
B01	Antithrombotic agents	60	56.6
A06	Drugs for constipation	48	45.3
C03	Diuretics	46	43.4
D02	Emollients and protectives	45	42.5
N06	Psychoanaleptics	43	40.6
A02	Drugs for acid related disorders	41	38.7
C09	Agents acting on the renin-angiotensin system	38	35.8
B03	Antianaemic preparations	29	27.4
C01	Cardiac therapy	26	24.5
N05	Psycholeptics	26	24.5
C07	Beta blocking agents	25	23.6
A12	Mineral supplements	24	22.6
H03	Thyroid therapy	24	22.6

### Potential for deprescribing

Among the residents with T2DM, a total of 346 PIMs were identified. The residents had from none to nine PIMs (Table 3), with a median number of three PIMs. In total, 96 residents (90.6 %) were prescribed at least one PIM. Frequency of PIMs by BNF classification is presented in Table 4. The most frequent PIMs were (1) statins prescribed without a valid indication (n = 50, 47.2 %); (2) more than one antihypertensive prescribed (n = 43, 40.6 %); (3) laxatives prescribed without a valid indication (n = 32, 30.2 %); (4) antidepressant prescribed without a valid indication (n = 32, 30.2 %); and (5) H2 blockers/proton pump inhibitors (PPI) prescribed without a valid indication (n = 27, 26.5 %).

**Table 3:** Total frequency of potentially inappropriate medicines in care home residents with Type 2 diabetes mellitus (n = 106)

PIMs	Residents	
n	n	%
0	10	9.4
1	17	16.0
2	12	11.3
3	21	19.8
4	18	17.0
5	13	12.3
6	6	5.7
7	4	3.8
8	4	3.8
9	1	0.9

*PIMs* potentially inappropriate medicines

**Table 4:** Frequency of potentially inappropriate medicines by classification of the British National Formulary, in residents with Type 2 diabetes mellitus (n = 106)

BNF chapter^a^	Number of criteria in chapter	Residents	
		n	%
Chapter 1—gastrointestinal system	4	70	20.2
Chapter 2—cardiovascular system	10	111	32.1
Chapter 3—respiratory system	3	1	0.3
Chapter 4—central nervous system	15	89	25.7
Chapter 5—infections	3	10	2.9
Chapter 6—bisphosphonates	1	9	2.6
Chapter 7—obstetrics, gynaecology and urinary tract disorders	5	7	2.0
Chapter 9—nutrition and blood	2	24	6.9
Chapter 10—musculoskeletal and joint diseases	4	13	3.8
Chapter 11—eye	1	0	0.0
Chapter 12—ear, nose and oropharynx	1	1	0.3
Chapter 13—skin	1	11	3.2
Total	50	346	100.0

*BNF* British National Formulary

^a^Chapters omitted indicated that these were not applicable to our population

Within the 20 % random sample chosen for validation by physician CG, a total of 67 PIMs were identified and 35 of these belonged to the top five frequent PIMs (Table 5). Out of the total of 67 PIMs the physician agreed that 26 of these could be discontinued without further question (38.8 %). A common example of this was statins without a valid indication. In the case of a further 40 PIMs (59.7 %) the physician indicated that medicine discontinuation should be considered, following access to other clinical data. An example here was to check blood pressure before deciding whether or not to discontinue excess antihypertensives. The physician recommended that one PIM (1.5 %) be changed to a different medicine. In this particular case, the combination of an SSRI with low-dose aspirin gave the resident an increased risk of gastrointestinal bleeding and hence the physician recommended keeping the ulcer prophylaxis, but replacing the H2 blocker with a proton pump inhibitor. None of the PIMs were considered for direct continuation.

**Table 5:** Validation of deprescribing potential for the top five frequently prescribed potentially inappropriate medicines

Description of PIM	Total population	Sample for validation	Validation category			
	n	n	Discontinue	Need more information	Change	Keep unchanged
Statin, no valid indication (107)^a^	50	12	12	0	0	0
Antihypertensive, more than one (105)^a^	43	7	0	7	0	0
Laxative, no valid indication (103b)^a^	32	7	0	7	0	0
Antidepressant, no valid indication (120a)^a^	32	4	0	4	0	0
H2 blocker/PPI, no valid indication (102)^a^	27	5	4	0	1	0
Total	184	35	16	18	1	0

*PIM* potentially inappropriate medicine, *PPI* proton pump inhibitor

^a^Numbers in parentheses indicate the assigned criteria number (Online resource 1)

## Discussion

This study found that UK care home residents with T2DM were younger and had a greater burden of active medical problems, prescriptions and polypharmacy than residents without diabetes. Using the NHS PrescQIPP document OSAMU, PIMs were identified for nine out of ten residents with T2DM, with the absence of a valid indication as the most common reason. Based on the available data, a physician with experience of care homes and medicines optimisation confirmed that 39 % of the PIMs could be directly discontinued, and acknowledged a potential for deprescribing in all but one of the remaining cases.

Our findings concur with previous studies showing that older persons with diabetes have higher rates of comorbidities [26] and prescriptions [5, 27, 28] compared to the general older population, thereby having increased risk for potentially inappropriate prescribing. The proportion of residents with at least one PIM is similar to that found for the general UK care home population when using a similar pragmatic approach for medicines review. The Northumbria Shine 2012 project, a prospective medicines optimisation study involving both clinicians and residents, used OSAMU as a resource in the shared decision making process [21]. When performing an extensive medicine review for 422 residents in 20 care homes in North Tyneside, UK, they found that 90.5 % of the residents required an intervention to their medicines [17, 21]. Stopping medicines was the most common intervention, required for seven out of ten residents [17, 21].

Failure to integrate comorbidities into clinical practice guidelines, and limited guidance on treatment for frail older patients are presented as leading reasons for the prescribing cascade so often seen in this population [29, 30]. Furthermore, frail elderly are normally excluded from randomised controlled trials and other robust studies that guidelines are built upon. Consequently, practitioners have little or no evidence-based guidance for how to prescribe for this vulnerable group of patients, and sometimes feel pressured to follow guidelines not developed based on the needs of these patients [30, 31].

It has been demonstrated that many medicines can be safely discontinued in older patients without causing adverse effects [11, 14, 17]. Still, concerns about withdrawal effects and lack of guidance on how and when to discontinue a medication discourage clinicians from attempting to do so [31, 32]. Several healthcare practitioners have expressed a need for deprescribing guidelines, especially for prevention-oriented medicines, as they may be less appropriate in the care home population [32]. In particular, statins have even been considered harmful in older patients, as low total cholesterol (<5.5 mmol/l) is associated with increased total mortality in those aged ≥80 years [18]. GPs sometimes choose not to follow recommended guidelines and refrain from prescribing statins in patients with T2DM. Questions about whether statins lead to improved quality of life, and concerns regarding frailty, multimorbidity and short life expectancy, are listed as the main reasons for this [33]. In our study, the physician who evaluated the PIMs agreed to stop all statins in the sample cases examined, for the same reasons.

In addition to evaluation of risk versus benefit of continued use of a medicine, the existence of a current indication is of particular concern for healthcare practitioners when considering deprescribing [32]. Four out of the five most common PIMs in our population involved medicines not having a valid indication. Similarly, no current indication was reported as the top reason for stopping medicines in the Northumbria Shine 2012 project [17], and according to Barber et al. [34] incomplete information in medical records is the prescribing error most frequently occurring in UK care homes. Many care homes receive prescribing services from multiple GPs, making clear and complete information crucial for adequate follow-up of the residents. A lack of information on indication may increase the potential for medication errors, and may also hamper deprescribing, as it adds to the uncertainty of whether the medicine is appropriate or not, especially if it is prescribed by a GP different to the one reviewing it. GPs often feel reluctant to change or stop medicines prescribed by colleagues, and also report to lack knowledge of geriatric pharmacotherapy [31].

In general, a lack of communication and team work between the GP practice, the pharmacy and the care home, and hence no integrated system for medicines management, is the reality for many UK care homes [34]. Appointing a lead GP for each care home and involving a pharmacist overseeing and regularly reviewing medicines use, are recommended to improve this [34]. Pharmacist involvement is valued by both GPs and care home staff [17] and can contribute to increased knowledge and awareness around medicines, as well as improve quality of medicine use [35]. The Northumbria Shine 2012 project demonstrated that a review process led by a prescribing pharmacist, where interventions were made available in the electronic medical notes for the GPs to challenge afterwards, was a cost-efficient approach. However, they debated that involving the GP during rather than after the review may result in even more interventions and greater savings [17]. This may be difficult to achieve at all care homes, and several clinical studies have shown that the GPs’ acceptance rate for medicine interventions suggested by pharmacists is generally high [17, 36, 37]. Although our approach was theoretical rather than clinical, the physician who evaluated the PIMs fully agreed with the pharmacist’s suggestions for deprescribing in 39 % of the cases, and acknowledged a potential for deprescribing in all but one of the remaining cases.

As this study was a cross-sectional and retrospective review of a selection of resident data from an RCT dataset, it has its limitations. For instance, we did not have information about the sequence of prescribing, information about duration of active medical problems, or previous medical problems and prescriptions. Neither did we have access to clinical data, such as blood pressure, lipids, weight and fluid intake. These data could have shed light on the appropriateness of even more therapies than we included as part of our analysis, and thus have facilitated a consideration of optimisation of therapy, not just the potential for deprescribing. We know from previous studies that blood glucose lowering therapy is not always optimal in the care home population [10, 11]. Additional clinical data could also have provided a better foundation for assessing the applicability of the criteria, and thus have given room for involving a more extensive team of clinicians to validate them. With a limited set of medical information, we identified 346 medicines as potentially inappropriate, where in a random sample a large proportion was directly endorsed for discontinuation by an experienced care home physician. If applied by clinical pharmacists or GPs with full access to all necessary medical information, maybe an even greater number of PIMs could have been identified and discontinued, and other therapies could also have been considered for optimisation.

We used a relatively new tool for evaluating appropriateness of medicines in the care home population. As such, comparison with other studies using other tools should be done with care. However, we have only compared our results to studies using similar, pragmatic approaches. In addition, more well-known tools, such as the STOPP/START criteria, have been considered less suitable when seeking to optimise drug therapy in the very frail old [13]. The tool used in this study is evidence-based, takes into account the complexity of care home residents and has proven to be efficient in this population [20]. Even though the sample size is small and performed in a limited geographical area, the resident population is comparable to that of other studies investigating different aspects of health status of care home residents both with and without DM in other parts of the UK [2, 38]. Hence, there is no reason to believe that the residents in this study are significantly different from the overall UK care home population.

The results of this study indicate that there is an unfulfilled potential for deprescribing in care home residents with T2DM. A more clinical approach with complete access to all relevant information and involvement of a team of clinicians, assessing relevant outcomes such as impact on glycaemic control and quality of life, should be the goal for future studies. It would be interesting to see if such a study gives similar results to those reported here. As a final note, when targeting care home medicines management, involvement of the resident should also be considered. Together with the best current research evidence and clinical expertise, the patient’s values and preferences make up the triad for evidence-based medicine [39].

## Conclusion

UK care home residents with T2DM have an increased burden of comorbidities, prescriptions and polypharmacy. Using an evidence-based, pragmatic medicines optimisation tool, we identified that the majority of these residents were prescribed at least one PIM. Validation of the PIMs by an experienced care home physician supports the clinical applicability of the ‘Optimising Safe and Appropriate Medicines Use’ document.

## Electronic supplementary material

Below is the link to the electronic supplementary material.

Supplementary material 1 (PDF 59 kb)

Supplementary material 2 (PDF 139 kb)
