# Supplementary material for: The potential for deprescribing in care home residents with Type 2 diabetes
Source: Int J Clin Pharm. 2016 May 30;38:977–84. doi: 10.1007/s11096-016-0323-4 (PMC4929175; doi:10.1007/s11096-016-0323-4)
Supplement: Supplementary file 1 — Supplementary material 1 (PDF 59 kb) [file 11096_2016_323_MOESM1_ESM.pdf]

Online Resource 1

Supplementary material for the paper *The potential for deprescribing in care home residents with type 2 diabetes*, by authors Lillan Mo Andreassen, Reidun Lisbet Skeide Kjome, Una Ørvim Sølvik, Julie Houghton, and James Antony Desborough.

Corresponding author: Lillan Mo Andreassen , M.Sc.Pharm, Department of Global Public Health and Primary Care, University of Bergen, Bergen, Norway. Email: Lillan.Andreassen@uib.no

Name of journal: International Journal of Clinical Pharmacy.

**Deprescribing criteria based on NHS PrescQIPP/UKMI document 'Optimising Safe and Appropriate Medicines Use' (OSAMU) [1]**	
BNF Chapter 1 – Gastrointestinal system	
Antispasmodics: How long have they been prescribed? Avoid long term use, highly anticholinergic preparations, uncertain effectiveness.	Criteria 101
H2 blockers / PPI: Check if there is a valid indication for prescribing e.g. is an NSAID still being taken? There has been no proven peptic ulcer, GI bleeding or dyspepsia for 1 year. Continued use may contribute to C. difficile infection.	Criteria 102
Laxatives: Previous use of opioid analgesics has reduced or stopped.<br>Regular bowel movements occur without difficulty. Patient is eating and drinking and has an adequate fluid intake.<br><br>If >1 laxatives are used, reduce and stop one at a time slowly. Do not stop treatment abruptly. Reduce stimulant laxative first, increase the dose of the osmotic laxative if necessary. Restart laxative if relapse occurs.	Criteria 103b<br><br><br><br>Criteria 103a
BNF Chapter 2 – Cardiovascular system	
Spironolactone: If dose >25 mg/day, the risk of hyperkalaemia is higher in older adults with heart failure, especially if taking an NSAID, ACE inhibitor, angiotensin II receptor blocker or potassium supplement.	Criteria 201

Antiarrhytmics: Rate control has better balance of benefits and harms than rhythm control for most older adults. Amiodarone is associated with multiple toxicities (thyroid, pulmonary, QT prolongation). Check all monitoring is being done.	Criteria 104
Antihypertensives – ACE inhibitors, beta blockers, angiotensin II receptor blockers, diuretics, calcium channel blockers: Check if there is a valid indication for prescribing, is the BP at a normal level or too low?<br><br>Do the known possible adverse drug reactions outweigh the possible benefits e.g. orthostatic hypotension, CNS effects, risk of falls; loop diuretic for ankle oedema – would compression hosiery be more appropriate?<br><br>If >1 antihypertensives are used, stop 1 at a time, maintaining the dose of the others without change. Restart antihypertensives if BP increases above 90 mmHg diastolic and/or 150 mmHg systolic (160 mmHg if no organ damage). Withdraw alpha agonist gradually to avoid severe rebound hypertension.	N/A<br><br>N/A<br><br>Criteria 105
Nitrates: The patient has not had chest pain for 6 months. The patient has reduced mobility.	Criteria 106
Statins / lipid lowering drugs: Re-evaluate the patients risk profile for primary and secondary prevention of cardiovascular disease – is there a valid indication for prescribing?<br><br>Stop in metastatic disease.	Criteria 107<br><br>N/A

Aspirin: Check if there is a valid indication for prescribing (e.g. re-evaluate the patients risk profile for primary prevention).<br>Do the known possible adverse drug reactions outweigh the possible benefits?<br><br>Is a dose of >150 mg/day being used for a cardiovascular indication?<br>Is aspirin being used for dizziness which is not clearly attributable to cerebrovascular disease?	Criteria 108<br><br><br><br>Criteria 202
Dipyridamole: Clopidogrel is now preferred over dipyridamole as more clinically and cost effective.	Criteria 109
Anticoagulants – oral and injected: Are LMWHs/oral anticoagulants prescribed following hip/knee replacement surgery still required? Stop warfarin if the risk of falls outweighs the benefits. Long term warfarin use (>6 months) is not recommended when the VTE was provoked by surgery, non-surgical trigger factors or the VTE occurred in the calf only.	N/A
Peripheral vasodilators: Check if there is a valid indication for prescribing. Clinical effectiveness often not established. Do the known possible adverse drug reactions outweigh the possible benefits?	N/A
Digoxin: Check if there is a valid indication for prescribing.<br>Do the known possible adverse drug reactions outweigh the possible benefits? E.g. if there is an increase in toxicity, decrease oral fluid intake.<br><br>Long term digoxin at >125 mcg/day in patient with impaired renal function can lead to an increased risk of toxicity.	Criteria 110<br><br><br><br><br>Criteria 203

BNF Chapter 3 – Respiratory system	
Theophylline: Monotherapy in COPD is not appropriate – safer, more effective alternatives are available.	Criteria 111
Oral corticosteroids: Prednisolone maintenance in COPD is not usually recommended. The magnitude and speed of dose reduction and withdrawal should be determined on a case by case basis. Gradual withdrawal should be considered for those who have received more than 3 weeks treatment, those who have received more than 40 mg prednisolone daily (or equivalent) or have other possible causes of adrenal suppression.	Criteria 112
Inhaled corticosteroids: In asthma – review every 3 months, has control been achieved, if yes; reduce dose slowly (by 50% every 3 months). In COPD – if an inhaled corticosteroid is not appropriate, a long acting abtimuscarinic bronchodilator can be used with a long acting beta2 agonist.	N/A
Antihistamines (first generation): Highly anticholinergic, clearance is reduced with advanced age, tolerance develops when used as a hypnotic, greater risk of confusion, dry mouth, constipation.	Criteria 113
BNF Chapter 4 – Central nervous system	
Chloral hydrate: Tolerance occurs within 10 days, risk outweighs benefits as overdose is only 3 times the recommended dose; avoid use, avoid prolonged use (and abrupt withdrawal thereafter).	Criteria 114
Meprobamate: High rate of physical dependence, very sedating, avoid use, avoid prolonged use, abrupt withdrawal may precipitate convulsions. EMEA recommended the suspensions of marketing authorisations in Jan 2012 as the risks of serious CNS side effects outweigh the benefits.	Criteria 115

Barbiturates: Intermediate acting preparations should only be used in severe intractable insomnia, avoid use in the elderly. High rate of physical dependence, tolerance to sleep benefits, risk of overdose at low doses.	Criteria 116
Benzodiazepines (including 'Z' drugs): Is use required if physical and psychological health and personal circumstances are stable? If the patient is willing, committed and compliant, and has adequate social support refer to a withdrawal clinic. Withdrawal should be gradual to avoid confusion, toxic psychosis and convulsions.<br><br>With long term use, risk of adverse effects including falls, exceeds therapeutic benefit of continued use.	N/A<br><br>Criteria 117a + 117b
Drugs for dementia: If MMSE <10, medicines may be continued if they help with behavior. NICE recommends memantine if MMSE <10. Review benefit, use should only continue if the MMSE score is ≥10 and treatment has an effect on the global, functional or behavioural symptoms.	N/A
Levodopa – carbidopa: Check if there is a valid indication for prescribing. Do the known possible adverse drug reactions outweigh the possible benefits?	Criteria 118
Antipsychotics: Check if there is a valid indication for prescribing.<br>Do the known possible adverse drug reactions outweigh the possible benefits? In dementia patients with behavioural and psychological symptoms, review and discontinue, particularly if there has been no response and symptoms are mild, unless there is extreme risk or distress for the patient. Standardized symptom evaluations and drug cessation attempts should be undertaken at regular intervals.<br><br>Are chlorpromazine or trifluoperazine being taken with other medicines that have anticholinergic activity and can increase risk of cognitive impairment e.g. TCADs, oxybutynin, chlorphenamine?	Criteria 119<br><br>Criteria 301

Antidepressants – Selective serotonin reuptake inhibitors (SSRIs), tricyclic antidepressants (TCADs), others e.g. MAOIs, agomelatine, duloxetine, reboxetine, venlafaxine, mirtazapine: Check if there is a valid indication for prescribing. For a single episode of depression treat for 6-9 months; for multiple episodes, treat for at least 2 years, no upper duration of treatment has been identified.<br><br>Dosulepin should not be prescribed.<br><br>Do the known possible adverse drug reactions outweigh the possible benefits? E.g. TCADs can worsen dementia, glaucoma, constipation, urinary retention; SSRIs may induce clinically significant hyponatremia.<br><br>Are TCADs being taken with other medicines that have anticholinergic activity and can increase risk of cognitive impairment e.g. chlorpromazine, oxybutynin, chlorphenamine? Reduce dose of antidepressants gradually to avoid withdrawal effects.	Criteria 120a<br><br>Criteria 120b<br><br>Criteria 302<br><br>Criteria 401
Opioid analgesics: Is a regular opioid still required? The risk of falls/constipation can outweigh the benefits. Consider non-drug options, switch to regular paracetamol.<br><br>Review laxatives.	Criteria 121a<br><br>Criteria 121b
Metoclopramide: Check if there is a valid indication for prescribing. How long has it been prescribed? Can cause extrapyramidal effects including tardive dyskinesia, risk greater in frail older adults.	Criteria 122

BNF Chapter 5 – Infections	
Antibacterials: Check if there is a valid indication for prescribing. Inappropriate uses – a bacterial infection has resolved; a viral infection has been diagnosed; prophylactic treatment prescribed but no pathogen isolated. Treatment of asymptomatic bacteriuria (ASB) in older patients and diabetes patients has no beneficial effects. There is a lack of evidence to evaluate the effect of preventing cathether associated ASB with antibiotics. Is fluid intake adequate?<br><br>Nitrofurantoin has potential for pulmonary toxicity, lack of efficacy in patients with CrCL <60ml/min due to inadequate drug concentration in the urine; avoid long term use.	Criteria 123a<br><br>Criteria 123b + N/A
Antifungals: Skin scrapings should be taken if systematic therapy is being considered or if there is doubt about the diagnosis. When a course of treatment of appropriate length has been finished, do not continue indefinitely e.g. oral and topical nystatin. For finger and toe nail infections, cure is achieved in only a minority of patients, the relapse rate is high.	Criteria 124
BNF Chapter 6 – Endocrine system	
Oestrogens ± progestogens: There is no mandatory limitation on the duration of HRT. Whether or not to continue therapy is dependent on an objective estimation on ongoing benefits and risks. Evidence of carcinogenic potential in breast and endometrium, lack of cardioprotective effect and cognitive protection in older women. Topical low dose oestrogen intravaginal cream safe and effective for dyspareunia and other vaginal symptoms.	N/A
Bisphosphonates: Check if there is a valid indication for prescribing.<br><br>Has treatment been taken for 5 years or more? Do the known possible adverse drug reactions outweigh the possible benefits? If the patient is at low risk of falls, are these still needed? Prolonged immobility is a risk factor for low BMD.	Criteria 125<br><br>N/A

BNF Chapter 7 – Obstetrics, gynaecology and urinary tract disorders	
Alpha blockers: Check if there is a valid indication for prescribing.<br><br>Use is generally not indicated if a patient has a long term (>2 months) catheter in situ.	Criteria 126<br><br>N/A
Antimuscarinics (for bladder/urinary tract symptoms): Check if there is a valid indication for prescribing. Review effectiveness after 3-6 months.<br><br>Check if continence pads are also used, is concomitant use necessary?<br><br>Do the known possible adverse drug reactions outweigh the possible benefits? E.g. postural hypotension, urinary retention, constipation.<br><br>Oxybutynin will decrease MMSE score in patients with dementia.<br><br>Are antimuscarinics being taken with other medicines that have anticholinergic activity and can increase risk of cognitive impairment e.g. chlorpromazine, TCADs, chlorphenamine?	Criteria 127<br><br>N/A<br><br>Criteria 402a<br><br>Criteria 402b<br><br>Criteria 303
**BNF Chapter 8 – Malignant disease and immunosuppression**	
Cytotoxics, immunosuppressants: What outcome is expected, do the known possible adverse drug reactions outweigh the possible benefits? Refer to doctor who initiated treatment.	N/A
**BNF Chapter 9 – Nutrition and blood**	
Sodium, potassium & iron supplements: Check if there is a valid indication for prescribing, do the known possible adverse drug reactions outweigh the possible benefits.	Criteria 130

Vitamins: Check if there is a valid indication for prescribing, e.g. does the patient have a disorder which requires vitamin & mineral supplements.	Criteria 131
Calcium + vitamin D: Does the patient have adequate levels through diet/sunlight exposure? If the patient is not mobile, is this still needed?	N/A
Sip feeds: Check if there is a valid indication for prescribing. Has a dietician recently reviewed the patient; is the patient able to prepare, or have someone else prepare fortified food and therefore does not need sip feeds.	N/A
BNF Chapter 10 – Musculoskeletal and joint diseases	
NSAIDs: Check if there is a valid indication for prescribing. Is an NSAID still needed/appropriate e.g. long term treatment of gout but no prophylaxis prescribed?<br><br>Do the known possible adverse drug reactions outweigh the possible benefits e.g. >3months use for symptom relief in mild osteoarthritis, use in patients with severe hypertension/heart failure/chronic renal failure.<br><br>If topical NSAIDs are continued indefinitely, review the need for use; short courses are generally advised.	Criteria 128a<br><br>Criteria 403<br><br>Criteria 128b
DMARDs: Discontinue penicillamine if there is no improvement within 1 year. Consider withdrawal of azathioprine and ciclosporin if there is no improvement within 3 months of use. Refer to doctor who initiated treatment.	N/A

Skeletal muscle relaxants: Often poorly tolerated because of anticholinergic adverse effects, sedation, risk of fracture, avoid use.	Criteria 129
TNF inhibitors: Psoriatic arthritis/Ankylosing spondylitis – discontinue adalimumab, etanercept and infliximab if there is inadequate response after 12 weeks. Rheumatoid arthritis/Juvenile idiopathic arthritis – withdraw adalimumab, etanercept and infliximab if response is not adequate within 6 months.	N/A
BNF Chapter 11 – Eye	
Eye drops/ointments: Review need for preservative free eye drops – is there a valid indication for prescribing (e.g. previous preservative toxicity), are eye drops instilled more than 4 times per day?<br><br>Have antibiotic preparations been continued without a review or stop date?	N/A<br><br>Criteria 132
BNF Chapter 12 – Ear, nose and oropharynx	
Drops, sprays, solutions etc.: Is the medicine still required? Have antibiotic / steroid / sympathomimetic preparations been continued without a review or stop date?	Criteria 133
BNF Chapter 13 – Skin	
Creams, ointments: Has the condition resolved and continued use may cause adverse effects or exacerbate the condition e.g. preparations containing antibacterials or corticosteroids?<br><br>Is the patient using sufficient emollient to avoid use of steroids or development of ulcers?	Criteria 134<br><br>N/A

Appendix 5 – Wound management products and elasticated garments	
Dressings: Wounds should be reviewed before prescribing to ensure correct dressing chosen. Chronic wounds change over time – refer difficult to treat wounds to a tissue viability nurse. Wounds should reduce in size over time. Address underlying problems e.g. soiling from incontinence, wrong choice of dressing etc. Larger dressings are more expensive than the smaller sizes. Query large size dressings on repeat prescriptions. Query quantities over 10 units per month, most dressings can stay in place for 3-5 days except on infected wounds, although some patients may have multiple wound sites. Avoid waste – prescribe the actual number of dressings needed rather than "1OP".	N/A

1. PrescQIPP. *Optimising Safe and Appropriate Medicines Use*, NHS PrescQIPP, UK Medicines Information, 2013.
