# Supplementary material for: The potential for deprescribing in care home residents with Type 2 diabetes
Source: Int J Clin Pharm. 2016 May 30;38:977–84. doi: 10.1007/s11096-016-0323-4 (PMC4929175; doi:10.1007/s11096-016-0323-4)
Supplement: Supplementary file 2 — Supplementary material 2 (PDF 139 kb) [file 11096_2016_323_MOESM2_ESM.pdf]

CODEBOOK FOR ANALYSIS

Supplementary material for the paper *The potential for deprescribing in care home residents with type 2 diabetes*, by authors Lillan Mo Andreassen, Reidun Lisbet Skeide Kjome, Una Ørvim Sølvik, Julie Houghton, and James Antony Desborough.

Corresponding author: Lillan Mo Andreassen , M.Sc.Pharm, Department of Global Public Health and Primary Care, University of Bergen, Bergen, Norway. Email: Lillan.Andreassen@uib.no

Name of journal: International Journal of Clinical Pharmacy.

Additional material to codebook:

Appendix 1: Generic names of medicines prescribed in our population. Each generic medicine has an assigned number that are used as a counting aid in our material. Numbers in parentheses following name of drug substances in the code book indicate the number that substance is given in our material.

Appendix 2: Anticholinergic medicines UK. All medicines with anticholinergic properties that are licensed in the UK. Based on a review paper, they are classified into high-potency anticholinergics and low-potency anticholinergics. Duran CE et al (2013). "Systematic review of anticholinergic risk scales in older adults." Eur J Clin Pharmacol 69(7): 1485-1496.

Appendix 3: List of anticholinergic medicines prescribed in our population. All medicines with anticholinergic properties prescribed in our population.

Appendix 4: Classified conditions, numbers and ICD. Table with an overview of all classified conditions and their accompanying ICD codes, as described in the CAREMED database by research staff. Each condition also has an assigned number that are used as a counting aid in our material.

All appendices can be found at the end of this document.

Category 1: Inappropriate choice of drug

101: Antispasmodics: Avoid long-term use, highly anticholinergic preparations, uncertain effectiveness.

Check antispasmodics use, Yes = 1, No = 0.

The following drugs are listed as antispasmodics in BNF:

Alverine citrate (6)
Atropine sulphate (not prescribed)
Dicycloverine hydrochloride (not prescribed)
Hyoscine butylbromide (93)
Mebeverine hydrochloride (124)
Peppermint oil (149)
Propantheline bromide (not prescribed)

102: H2 blockers / PPI: Check if there is a valid indication for prescribing e.g. NSAID still being taken, diagnosis of peptic ulcer, GI bleeding or dyspepsia. Continued use may contribute to C. difficile infection.

Check for valid indication for use of H2 blockers / PPI; No valid indication = 1, Valid indication = 0.

The following diagnoses have by the researchers been identified as valid indications:

Barrett's oesophagus (20)
Gastric haemorrhage (70)
Gastro-oesophagal reflux (73)
Hiatus hernia (88)
Indigestion (dyspepsia) (98)
Oesophagitis (134)

103: Laxatives: Check if there is a valid indication for prescribing e.g. opioid analgesics still being taken, diagnosis of constipation. Also check if >1 laxative is being used.

103a: *Check if there is more than one laxative being prescribed; Yes = 1, No = 0.*

103b: *Check for valid indication for use of laxatives; No valid indication = 1, Valid indication = 0.*

Prescribed opioid analgesic (N02A) OR the diagnosis of constipation or other diagnosis that could justify use. Disorders affecting colon, like diverticular disease, cancer, hernia of colon etc. have by the researchers been identified as valid indications.

104: Antiarrhytmics: Amiodarone is associated with multiple toxicities (thyroid, pulmonary, QT prolongation), should not be prescribed.

Check for use of amiodarone; Yes = 1, No = 0.

105: Antihypertensives - ACE inhibitors, beta blockers, A2RB, diuretics, calcium channel blockers: Check if >1 antihypertensive is being used.

Check if there is more than one antihypertensive agent being prescribed; Yes = 1, No = 0.

106: Nitrates: Check if there is a valid indication for prescribing e.g. chest pain/angina.

Check for valid indication for use of nitrates; No valid indication = 1, Valid indication = 0.

The following diagnoses have by the researchers been identified as valid indications:

Angina pectoris
Heart failure
Ischaemic heart disease (IHD)
Myocardial infarction

107: Statins / lipid lowering drugs: Re-evaluate the patients risk profile for primary and secondary prevention of cardiovascular disease – is there a valid indication for prescribing? Not sufficient data to recommend in the population aged 80+, with or without CVD (Petersen et al 2010).

Check for valid indication for use of statins; No valid indication = 1, Valid indication = 0.

108: Aspirin: Check if there is a valid indication for prescribing e.g. re-evaluate the patients risk profile for primary prevention. Do the known possible adverse drug reactions (risk of bleeding) outweigh the possible benefits (cardiovascular endpoints)? Recent studies on patients with high baseline risk, such as those with T2DM, have not found the expected benefits of aspirin on cardiovascular endpoint, and elderly patients are also more vulnerable to major haemorrhage.

Check for valid indication for use of aspirin; No valid indication = 1, Valid indication = 0.

The following diagnoses have by the researchers been identified as valid indications for secondary prevention:

Angina pectoris
Atrial fibrillation and flutter
Aortic valve disorder
Cerebrovascular disease
DVT
Heart failure
Ischaemic heart diseases (IHD)
Myocardial infarction
Stroke
Transient cerebral ischaemic attack

109: Dipyridamole: Clopidogrel is now preferred over dipyridamole as more clinically and cost effective.

Check for use of dipyridamole; Yes = 1, No = 0.

110: Digoxin: Check if there is a valid indication for prescribing, e.g. heart failure or arrhythmias.

Check for valid indication for use of digoxin; No valid indication = 1, Valid indication = 0.

The following diagnoses have by the researchers been identified as valid indications:

Atrial fibrillation/flutter
Heart failure
Pulmonary embolism
Pulmonary oedema

111: Theophylline: monotherapy in COPD is not appropriate – safer, more effective alternatives available.

Check for theophylline as monotherapy in COPD; Yes = 1, No = 0.

112: Oral corticosteroids: Prednisolone maintenance in COPD is not usually recommended. Gradual withdrawal should be considered for those who have received more than 3 weeks treatment, those who have received more than 40 mg prednisolone daily (or equivalent) or have other possible causes of adrenal suppression.

Check for prednisolone as long term therapy (>3 weeks) in COPD; Yes = 1, No = 0.

Long term use (>3 weeks) of prednisolone (or daily prednisolone doses >40 mg daily).

113: Antihistamines (first generation): Highly anticholinergic, clearance is reduced with advanced age, tolerance develops when used as a hypnotic, greater risk of confusion, dry mouth, constipation.

Check for use of first generation antihistamines; Yes = 1, No = 0.

The following drugs are listed as antihistamines (first generation) in BNF:

Alimemazine tartrate
Chlorphenamine maleate
Cinnarizine
Clemastine
Cyclizine
Cyproheptadine hydrochloride
Hydroxyzine hydrochloride
Ketotifen
Perphenazine
Prochlorperazine

Promethazine hydrochloride/promethazine teoclate
Trifluoperazine

114: Chloral hydrate: Tolerance occurs within 10 days, risk outweighs benefits as overdose is only 3 times the recommended dose; avoid use, avoid prolonged use (and abrupt withdrawal thereafter).

Check for use of chloral hydrate; Yes = 1, No = 0.

115: Meprobamate: High rate of physical dependence, very sedating, avoid use, avoid prolonged use, abrupt withdrawal may precipitate convulsions. EMEA recommended the suspensions of marketing authorisations in Jan 2012 as the risks of serious CNS side effects outweigh the benefits.

Check for use of chloral hydrate; Yes = 1, No = 0.

116: Barbiturates: Intermediate acting preparations should only be used in severe intractable insomnia, avoid use in the elderly. High rate of physical dependence, tolerance to sleep benefits, risk of overdose at low doses.

Check for use of intermediate acting barbiturates; Yes = 1, No = 0.

117: Benzodiazepines (including 'Z' drugs): With long term use, risk of adverse effects including falls, exceeds therapeutic benefit of continued use.

117a: *Check for benzodiazepines as long term therapy (>3 months); Yes = 1, No = 0.*

117b: *Check for 'Z' drugs as long term therapy (>3 months); Yes = 1, No = 0.*

118: Levodopa – carbidopa: Check if there is a valid indication for prescribing, i.e. Parkinson's disease.

Check for valid indication for use of digoxin; No valid indication = 1, Valid indication = 0.

119: Antipsychotics: Check if there is a valid indication for prescribing. Do the known possible adverse drug reactions outweigh the possible benefits? In dementia patients with behavioural and psychological symptoms, review and discontinue, particularly if there has been no response and symptoms are mild, unless there is extreme risk or distress for the patient. Standardized symptom evaluations and drug cessation attempts should be undertaken at regular intervals.

Schizophrenia or other diagnoses that can justify use (e.g. delirium, agitation, hallucination, dementia). For other diagnoses than schizophrenia, the prescription should be PRN/short term to be justified.

120: Antidepressants – Selective serotonin reuptake inhibitors (SSRIs), tricyclic antidepressants (TCADs), others e.g. MAOIs, agomelatine, duloxetine, reboxetine,

venlafaxine, mirtazapine: Check if there is a valid indication for prescribing, e.g. depression. Dosulepin should not be prescribed.

120a: *Check for valid indication for use of antidepressants; No valid indication = 1, Valid indication = 0.*

Valid indication: Depression.

120b: *Check for use of dosulepin; Yes = 1, No = 0.*

121: Opioid analgesics: Is a regular opioid still required? The risk of falls/constipation can outweigh the benefits. Consider non-drug options, switch to regular paracetamol. Review laxatives.

121a: *Check for justified use of regular opioids; No diagnosis justifying use = 1, Diagnosis justifying use = 0.*

Where regular use is documented, check if use may be justified AND if paracetamol is also prescribed. The following diagnoses have by the researchers been identified (in this population) as valid indications of regular opioid use:

Gout
Osteoarthritis
Osteoporosis / Paget’s disease
Sudek’s atrophy

121b: *Check for prescription of laxative in residents prescribed regular opioids, No laxative = 1, Laxative = 0.*

122: Metoclopramide: Check if there is a valid indication for prescribing. How long has it been prescribed? Can cause extrapyramidal effects including tardive dyskinesia, risk greater in frail older adults.

Check for valid indication for use of metoclopramide; No valid indication = 1, Valid indication = 0.

The following diagnoses have by the researchers been identified as valid indications:

Cancer
GI disorders (not specified)
Migraine

123: Antibacterials: Check if there is a valid indication for prescribing. Inappropriate uses – a bacterial infection has resolved; a viral infection has been diagnosed; prophylactic treatment prescribed but no pathogen isolated. Treatment of asymptomatic bacteriuria (ASB) in older patients and diabetes patients has no beneficial effects. Nitrofurantoin has potential for pulmonary toxicity; avoid long term use.

123a: *Check for valid indication for use of antibacterials; No valid indication = 1, Valid indication = 0.*

Diagnoses that can justify use: e.g. bacterial infections, or conditions putting resident at risk of bacterial infection.

123b: *Check for long term use (>3 weeks) of nitrofurantoin; Over 3 weeks = 1, Under 3 weeks = 0.*

124: Antifungals: When a course of treatment of appropriate length has been finished, do not continue indefinitely e.g. oral and topical nystatin.

Check for long term use (>3 weeks) of antifungals with no valid indication; No valid indication = 1, Valid indication = 0.

Check for long term use (>3 weeks) AND no valid indication in medical records. The following drugs are listed as antifungals in BNF:

Amorolfine
Amphotericin
Benzoic acid
Caspofungin
Clotrimazole (52 + 90)
Econazole nitrate
Fluconazole
Flucytosine
Griseofulvin
Itrakonazole
Ketoconazole (106)
Miconazole nitrate (130 + 91)
Nystatin (139)
Posaconazole
Salicylic acid
Suloconazole nitrate
Terbinafine
Tioconazole
Undecenoates
Voriconazole

125: Bisphosphonates: Check if there is a valid indication for prescribing.

Check for valid indication for use of bisphosphonates; No valid indication = 1, Valid indication = 0.

The following diagnoses have by the researchers been identified as valid indications:

Osteoporosis
Paget’s disease of bone

126: Alpha blockers: Check if there is a valid indication for prescribing.

Check for valid indication for use of alpha blockers; No valid indication = 1, Valid indication = 0.

The following diagnoses have by the researchers been identified as valid indications:

Hyperplasia of prostate
Overactive bladder

127: Antimuscarinics (for bladder/urinary tract symptoms): Check if there is a valid indication for prescribing.

Check for valid indication for use of antimuscarinics for bladder/urinary tract symptoms; No valid indication = 1, Valid indication = 0.

The following diagnoses have by the researchers been identified as valid indications:

Incontinence
Overactive bladder

128: NSAIDs: Check if there is a valid indication for prescribing. Is an NSAID still needed/appropriate e.g. long term treatment of gout but no prophylaxis prescribed? If topical NSAIDs are continued indefinitely, review the need for use; short courses are generally advised.

128a: *Check for valid indication for use of oral NSAIDs; No valid indication = 1, Valid indication = 0.*

NB! If used for long-term treatment, GI prophylaxis should also be prescribed! The following diagnoses have by the researchers been identified as valid indications:

Gout
Musculoskeletal diseases (not specified)

128b: *Check for topical NSAIDs as long term therapy (>3 months); Yes = 1, No = 0.*

Preparations prescribed prn are considered short term use.

129: Skeletal muscle relaxants: Often poorly tolerated because of anticholinergic adverse effects, sedation, risk of fracture, avoid use.

Check use of skeletal muscle relaxants; Yes = 1, No = 0. (even if valid indication!)

The following drugs are listed as skeletal muscle relaxants in BNF:

Baclofen (19)
Carisoprodol (not prescribed)

Dantrolene (not prescribed)
Diazepam (not prescribed)
Methacarbamol (not prescribed)
Quinine (164)
Tizanidine (not prescribed)

130: Sodium, potassium & iron supplements: Check if there is a valid indication for prescribing.

Check for valid indication for use of sodium, potassium & iron supplements; No valid indication = 1, Valid indication = 0.

The following diagnoses have by the researchers been identified as valid indications:

Anaemia
Vitamin B12 deficiency

131: Vitamins: Check if there is a valid indication for prescribing, e.g. does the patient have a disorder which requires vitamin & mineral supplements.

Check for valid indication for use of vitamins; No valid indication = 1, Valid indication = 0.

Valid indication: ‘Vitamin/mineral deficiency’ or use of methotrexate if receiving folic acid.

132: Eye drops/ointments: Have antibiotic preparations been continued without a review or stop date?

Check for long term use of antibiotic preparations without valid indication; No valid indication = 1, Valid indication = 0.

Valid indication: Bacterial infections.

133: Ear, nose and oropharynx: Drops, sprays, solutions etc.: Have antibiotic / steroid / sympathomimetic preparations been continued without a review or stop date?

Check for long term use of antibiotic / steroid / sympathomimetic preparations without valid indication; No valid indication = 1, Valid indication = 0.

Valid indication: Bacterial infections etc.

134: Skin: Creams, ointments: Has the condition resolved and continued use may cause adverse effects or exacerbate the condition e.g. preparations containing antibacterials or corticosteroids?

Check for antibiotic / steroid preparations without valid indication; No valid indication = 1, Valid indication = 0.

Valid indication: Bacterial infections, skin disorders.

Category 2: Inappropriate dosage of drugs

201: Spironolactone: If dose >25 mg/day, the risk of hyperkalaemia is higher in older adults with heart failure.

Check for dose of spironolactone >25mg/day in residents with heart failure; Yes = 1, No = 0.

202: Aspirin: Is a dose of >150 mg/day being used for a cardiovascular indication?

Check for dose of aspirin >150mg/day; Yes = 1, No = 0.

203: Digoxin: Long-term digoxin at >125 mcg/day in patient with impaired renal function can lead to an increased risk of toxicity.

Check for dose of digoxin >125mcg/day in residents with renal failure; Yes = 1, No = 0.

Category 3: Inappropriate drug-drug combinations

301: Antipsychotics: Are chlorpromazine or trifluoperazine being taken with other medicines that have anticholinergic activity and can increase risk of cognitive impairment e.g. TCADs, oxybutynin, chlorphenamine?

Check for combination of antipsychotics (on anticholinergic list) with other anticholinergic drugs; Yes = 1, No = 0.

Antipsychotics with anticholinergic effect according to list (Appendix 2):

Chlorpromazine
Clozapine
Fluphenazine
Haloperidol
Levomepromazine
Lithium
Olanzapine (140)
Pimozide
Prochlorperazine
Promazine
Quetiapine (163)
Risperidone (167)

302: Antidepressants: Are TCADs being taken with other medicines that have anticholinergic activity and can increase risk of cognitive impairment e.g. chlorpromazine, oxybutynin, chlorphenamine? Reduce dose of antidepressants gradually to avoid withdrawal effects.

Check for combination of antidepressants (on anticholinergic list) with other anticholinergic drugs; Yes = 1, No = 0.

Antidepressants with anticholinergic effect according to list (Appendix 2):

Amitriptyline (10)
Citalopram (46)
Clomipramine
Dosulepin (65)
Doxepin
Fluoxetine (78)
Fluvoxamine
Imipramine
Mirtazapine (131)
Nortriptyline
Paroxetine (148)
Phenelzine
Trazodone (186)
Trimipramine

303: Antimuscarinics (for bladder/urinary tract symptoms): Are antimuscarinics being taken with other medicines that have anticholinergic activity and can increase risk of cognitive impairment e.g. chlorpromazine, TCADs, chlorphenamine?

Check for combination of antimuscarinics for bladder/urinary tract symptoms (on anticholinergic list) with other anticholinergic drugs; Yes = 1, No = 0.

Antimuscarinics with anticholinergic effect according to list (Appendix 2):

Darifenacin
Flavoxate
Oxybutynin (144)
Tolterodine (184)

Category 4: Inappropriate drug-disease combinations

401: Antidepressants: Do the known possible adverse drug reactions outweigh the possible benefits? E.g. TCADs can worsen dementia, glaucoma, constipation, urinary retention; SSRIs may induce clinically significant hyponatremia.

Check for use of TCADs in residents with diagnoses of dementia, glaucoma, constipation or urinary retention; Yes = 1, No = 0.

The following diagnoses have been checked for:

Constipation
Dementia
Glaucoma
Urinary retention (or related diagnoses, e.g. BPH)

402: Antimuscarinics (for bladder/urinary tract symptoms): Do the known possible adverse drug reactions outweigh the possible benefits? E.g. postural hypotension, urinary retention, constipation. Oxybutynin will decrease MMSE score in patients with dementia.

402a: *Check for use of antimuscarinics for bladder/urinary tract symptoms in residents with diagnoses of hypotension, urinary retention or constipation; Yes = 1, No = 0.*

The following diagnoses have been checked for:

Constipation
Hypotension
Urinary retention (or related diagnoses, e.g. BPH)

402b: *Check for use of oxybutynin in residents with dementia; Yes = 1, No = 0.*

403: NSAIDs: Do the known possible adverse drug reactions outweigh the possible benefits e.g. use in patients with severe hypertension/heart failure/chronic renal failure.

Check for use of oral NSAIDs in residents with heart failure or chronic renal failure; Yes = 1, No = 0.

OSAMU criteria not applied

Antihypertensives - ACE inhibitors, beta blockers, A2RB, diuretics, calcium channel blockers: Check if there is a valid indication for prescribing, is the BP at a normal level or too low? Do the known possible adverse drug reactions outweigh the possible benefits e.g. orthostatic hypotension, CNS effects, risk of falls, loop diuretic for ancle oedema – would compression hosiery be more appropriate?

Limited access to clinical data, need to make too many assumptions to evaluate.

Statins: Stop in metastatic disease.

Do not have access to data that can tell whether resident has metastatic disease.

Anticoagulants – oral and injected: Are LMWHs/oral anticoagulants prescribed following hip/knee replacement surgery still required? Stop warfarin if the risk of falls outweighs the benefits. Long term warfarin use (>6 months) is not recommended when

the VTE was provoked by surgery, non-surgical trigger factors or the VTE occurred in the calf only.

Limited access to clinical data, unable to evaluate.

Peripheral vasodilators: Check if there is a valid indication for prescribing. Clinical effectiveness often not established. Do the known possible adverse drug reactions outweigh the possible benefits?

Peripheral vasodilators are not prescribed in our population.

Inhaled corticosteroids: In asthma – review every 3 months, has control been achieved, if yes; reduce dose slowly (by 50% every 3 months). In COPD – if an inhaled corticosteroid is not appropriate, a long acting abtimuscarinic bronchodilator can be used with a long acting beta2 agonist.

No access to clinical data that is needed to evaluate this.

Benzodiazepines (including 'Z' drugs): Is use required if physical and psychological health and personal circumstances are stable? If the patient is willing, committed and compliant, and has adequate social support, refer to a withdrawal clinic.

No access to the information necessary to evaluate this.

Drugs for dementia: If MMSE <10, medicines may be continued if they help with behavior. NICE recommends memantine if MMSE <10. Review benefit, use should only continue if the MMSE score is ≥10 and treatment has an effect on the global, functional or behavioural symptoms.

No access to MMSE scores.

Antibacterials: Nitrofurantoin, lack of efficacy in patients with CrCl <60 ml/min due to inadequate drug concentration in the urine.

No access to clinical data that is needed to evaluate this.

Oestrogens ± progestogens: There is no mandatory limitation on the duration of HRT. Whether or not to continue therapy is dependent on an objective estimation on ongoing benefits and risks. Evidence of carcinogenic potential in breast and endometrium, lack of cardioprotective effect and cognitive protection in older women. Topical low dose oestrogen intravaginal cream safe and effective for dyspareunia and other vaginal symptoms.

Not complete access to all information needed to make this evaluation.

Bisphosphonates: Has treatment been taken for 5 years or more? Do the known possible adverse drug reactions outweigh the possible benefits? If the patient is at low risk of falls, are these still needed? Prolonged immobility is a risk factor for BMD.

Do not have access to information necessary to evaluate this.

Alpha blockers: Use is generally not indicated if a patient has a long term (>2 months) catheter in situ.

Do not have access to information necessary to evaluate this.

Antimuscarinics (for bladder/urinary tract symptoms): Check if continence pads are also used, is concomitant use necessary?

Do not have access to information necessary to evaluate this.

Cytotoxics, immunosuppressants: What outcome is expected, do the known possible adverse drug reactions outweigh the possible benefits? Refer to doctor who initiated treatment.

Do not have access to information necessary to evaluate this.

Calcium + vitamin D: Does the patient have adequate levels through diet/sunlight exposure? If the patient is not mobile, is this still needed?

Do not have access to information necessary to evaluate this.

Sip feeds: Check if there is a valid indication for prescribing. Has a dietician recently reviewed the patient; is the patient able to prepare, or have someone else prepare fortified food and therefore does not need sip feeds.

Do not have access to information necessary to evaluate this.

DMARDs: Discontinue penicillamine if there is no improvement within 1 year. Consider withdrawal of azathioprine and ciclosporin if there is no improvement within 3 months of use. Refer to doctor who initiated treatment.

Do not have access to information necessary to evaluate this.

TNF inhibitors: Psoriatic arthritis/Ankylosing spondylitis – discontinue adalimumab, etanercept and infliximab if there is inadequate response after 12 weeks. Rheumatoid arthritis/Juvenile idiopathic arthritis – withdraw adalimumab, etanercept and infliximab if response is not adequate within 6 months.

Do not have access to information necessary to evaluate this.

Eye drops/ointments: Review need for preservative free eye drops – is there a valid indication for prescribing (e.g. previous preservative toxicity), are eye drops instilled more than 4 times per day?

Do not have access to information necessary to evaluate this.

Creams, ointments: Is the patient using sufficient emollient to avoid use of steroids or development of ulcers?

Do not have access to information necessary to evaluate this.

Dressings: Wounds should be reviewed before prescribing to ensure correct dressing chosen. Chronic wounds change over time – refer difficult to treat wounds to a tissue viability nurse. Wounds should reduce in size over time. Address underlying problems e.g. soiling from incontinence, wrong choice of dressing etc. Larger dressings are more expensive than the smaller sizes. Query large size dressings on repeat prescriptions. Query quantities over 10 units per month, most dressings can stay in place for 3-5 days except on infected wounds, although some patients may have multiple wound sites. Avoid waste – prescribe the actual number of dressings needed rather than "1OP".

Not relevant.

Appendix 1: Generic names of medicines prescribed in our population

1	Alendronic acid
2	Alfuzosin hydrochloride
3	Alginate
4	Aliskiren
5	Allopurinol
6	Alverine citrate
7	Amiloride + furosemide
8	Amiodarone
9	Amisulpride
10	Amitriptyline
11	Amlodipine
12	Amoxicillin
13	Amoxicillin + clavulanic acid
14	Anastrozole
15	Ascorbic acid (vit C)
16	Aspirin
17	Atenolol
18	Atorvastatin
19	Baclofen
20	Barrier preparation
21	Beclomethasone dipropionate
22	Beclomethasone + formoterol
23	Bendroflumethiazide
24	Benzerazide hydrochloride + levodopa
25	Betamethasone dipropionate
26	Betamethasone valerate
27	Betamethasone valerate + fusidic acid
28	Bimatoprost
29	Bisoprolol
30	Brimonidine
31	Budesonide
32	Bumetanide
33	Buprenorphine
34	Calcium+vitD
35	Calcipotriol + betamethasone
36	Candesartan
37	Carbidopa + levodopa
38	Carbimazole
39	Carbomer 980 (eye lubricant)
40	Carmellose sodium (eye lubricant)
41	Carvedilol
42	Cefradine
43	Cetirizine
44	Cinchocaine hydrochloride + fluocortolone
45	Cinchocaine hydrochloride + prednisolone
46	Citalopram
47	Clobetasol propionate
48	Clobetasone butyrate
49	Clonazepam
50	Clopidogrel
51	Cloral betaine
52	Clotrimazole
53	Coal tar
54	Codeine + paracetamol
55	Codeine
56	Crotamiton
57	Diclofenac
58	Digoxin
59	Dihydrocodeine tartrate
60	Diltiazem
61	Dipyridamole
62	Disodium etidronate
63	Domperidone
64	Donepezil
65	Dosulepin hydrochloride
66	Doxazosin
67	Emollient
68	Enalapril maleate
69	Felbinac
70	Fentanyl
71	Ferrous fumarate
72	Ferrous gluconate
73	Ferrous sulphate
74	Fexofenadine
75	Finasteride
76	Flecainide
77	Fluticasone propionate
78	Fluoxetine
79	Folic acid
80	Foods
81	Furosemide
82	Gabapentin
83	Galantamine
84	Gliclazide
85	Glimepiride

86	Glipizide
87	Glucose
88	Glyceryl trinitrate
89	Hydrocortisone topical
90	Hydrocortisone + clotrimazole topical
91	Hydrocortisone + miconazole topical
92	Hydroxocobalamin (vit B12)
93	Hyoscine
94	Hypromellose (eye lubricant)
95	Ibuprofen
96	Indoramin
97	Insulin (human)
98	Insulin aspart
99	Insulin detemir
100	Insulin glargine
101	Insulin lispro
102	Ipratropiumbromid
103	Isosorbide dinitrate
104	Isosorbide mononitrate
105	Isphagula husk
106	Ketokonazole (coal tar) shampoo
107	Ketoprofen
108	Lactulose
109	Lansoprazole
110	Latanoprost
111	Latanoprost + timolol
112	Lercanidipine
113	Leuprorelin
114	Levetiracetam
115	Levothyroxine
116	Liquid paraffin
117	Liquid paraffin (eye lubricant)
118	Lisinopril
119	Loperamide
120	Loratadine
121	Lorazepam
122	Macrogol
123	Magnesium salt + liquid paraffin
124	Mebeverine hydrochloride
125	Meptazinol
126	Metformin
127	Methotrexate
128	Metoclopramide
129	Metoprolol tartrate
130	Miconazole

131	Mirtazapine
132	Mometasone furoate
133	Morphine
134	Multivitamin
135	Nicorandil
136	Nifedipine
137	Nitrazepam
138	Nitrofurantoin
139	Nystatin
140	Olanzapine
141	Olive oil
142	Olmesartan
143	Omeprazole
144	Oxybutynin
145	Oxycodone
146	Pantoprazole
147	Paracetamol
148	Paroxetine
149	Peppermint oil
150	Pericyazine
151	Perindopril
152	Permethrin
153	Phenytoin
154	Pioglitazone
155	Piroxicam topical
156	Polyvinyl alcohol (eye lubricant)
157	Potassium chloride
158	Pravastatin
159	Prednisolone
160	Procyclidine
161	Propranolol
162	Propylthiouracil
163	Quetiapine
164	Quinine
165	Ramipril
166	Ranitidine
167	Risperidone
168	Salbutamol
169	Salmeterol
170	Senna
171	Silver sulfadiazine
172	Simvastatin
173	Sodium chloride topical
174	Sodium citrate rectal
175	Sodium valproate

176	Sotalol
177	Spironolactone
178	Sterculia
179	Tamsulosin
180	Temazepam
181	Theophylline
182	Tiotropium
183	Tolbutamide
184	Tolterodine
185	Tramadol hydrochloride

186	Trazodone
187	Trimethoprim
188	Venlafaxine
189	Warfarin sodium
190	Zuclopenthixol
191	Zinc sulphate
192	Zolpidem
193	Zopiclone
194	Missing

Appendix 2: Anticholinergic medicines UK

High-potency anticholinergics

Generic name	ATC code
Amitriptyline	N06AA09
Atropine	A03BA01
Belladonna alkaloids	A03BA04
Benzatropine	N04AC01
Chlorphenamine	R06AB04
Chlorpromazine	N05AA01
Clemastine	R06AA04
Clomipramine	N06AA04
Clozapine	N05AH02
Cyproheptadine	R06AX02
Darifenacin	G04BD10
Dicyclomine/Dicycloverine	A03AA07
Diphenhydramine	R06AA02
Doxepin	N06AA12
Flavoxate	G04BD02
Fluphenazine	N05AB02
Homatropine	S01FA05
Hydroxyzine	N05BB01
Imipramine	N06AA02
Ipratropium	R03BB01
Levomepromazine	N05AA02
Nortriptyline	N06AA10
Orphenadrine	N04AB02
Oxybutynin	G04BD07
Procyclidine	N04AA04
Promethazine	R06AD02
Propantheline	A03AB05
Scopolamine (Hyoscine)	A04AD01
Tizanidine	M03BX02
Tolterodine	G04BD07
Trihexylphenidyl	N04AA01
Trimipramine	N06AA06

Low-potency anticholinergics

Generic name	ATC code
Alimemazine	R06AD01
Amantadine	N04BB01
Baclofen	M03BX01
Bromocriptine	N04BC01
Carbamazepine	N03AF01
Cetirizine	R06AE07
Chlordiazepoxide	N05BA02
Cimetidine	A02BA01
Citalopram	N06AB04
Clonazepam	N03AE01
Codeine	R05DA04
Diazepam	N05BA01
Digitoxin	C01AA04
Disopyramide	C01BA03
Domperidone	A03FA03
Dosulepin	N06AA16
Entacapone	N04BX02
Fentanyl	N02AB03
Fexofenadine	R06AX26
Fluoxetine	N06AB03
Fluvoxamine	N06AB08
Haloperidol	N05AD01
Ketorolac	M01AB15
Lithium	N05AN01
Loperamide	A07DA03
Loratadine	R06AX13
Methadone	N07BC02
Methocarbamol	M03BA03
Mirtazapine	N06AX11
Morphine	N02AA01
Olanzapine	N05AH03
Oxcarbazepine	N03AF02
Oxycodone	N02AA05
Paroxetine	N06AB05
Phenelzine	N06AF03
Pimozide	N05AG02
Prochlorperazine	N05AB04
Promazine	N05AA03
Quetiapine (fumarate)	N05AH04
Ranitidine	A02BA02
Risperidone	N05AX08
Temazepam	N05CD07
Theophylline	R03DA04
Tramadol	N02AX02
Trazodone	N06AX05

Both lists based on Duran CE et al (2013). "Systematic review of anticholinergic risk scales in older adults." Eur J Clin Pharmacol 69(7): 1485-1496.

Appendix 3: List of anticholinergic medicines prescribed in our population

No	Generic name	Potency
10	Amitriptyline	H
19	Baclofen	L
43	Cetirizine	L
46	Citalopram	L
49	Clonazepam	L
54	Codeine + paracetamol	L
55	Codeine	L
63	Domperidone	L
65	Dosulepin hydrochloride	L
70	Fentanyl	L
74	Fexofenadine	L
78	Fluoxetine	L
93	Hyoscine	H
102	Ipratropiumbromid	H
119	Loperamide	L
120	Loratadine	L
131	Mirtazapine	L
133	Morphine	L
140	Olanzapine	L
144	Oxybutynin	H
145	Oxycodone	L
148	Paroxetine	L
160	Procyclidine	H
163	Quetiapine	L
166	Ranitidine	L
167	Risperidone	L
180	Temazepam	L
181	Theophylline	L
184	Tolterodine	H
185	Tramadol hydrochloride	L
186	Trazodone	L

Appendix 4: Classified conditions numbers and ICD

No	Name	ICD 1	ICD 2	ICD 3	No of residents with diagnosis
1	Cancer (neoplasms - other)				0
2	Abnormal weight loss	R00-R99			1
3	Acute bronchitis				0
4	Aggressive personality				0
5	Alcohol misuse				0
6	Allergic rhinitis				0
7	Alzheimer's disease	G00-G99	G30-G32	G30	10
8	Anaemia	D50-D89			12
9	Angina	I00-I99	I20-I25	I20	7
10	Anxiety	F00-F99	F40-F48		5
11	Anxiety with depression	F00-F99	F40-F48		2
12	Aortic aneurysm	I00-I99	I70-I79	I71	1
13	Aortic valve disorder	I00-I99	I30-I52		2
14	Apnoea	G00-G99	G40-G47		1
15	Arteritis				0
16	Arthropathy	M00-M99	M00-M25		1
17	Asthma	J00-J99	J40-J47	J45	5
18	Atrial fibrillation and flutter	I00-I99	I30-I52	I48	25
19	Back pain (dorsalgia)	M00-M99	M40-M54	M54	5
20	Barrett's oesophagus	K00-K93	K20-K31	K22	2
21	Behavioural management				0
22	Bone pain				0
23	Bronchiectasis				0
24	Bronchitis (recurrent)				0
25	Cancer (neoplasms - benign)				0
26	Cancer (neoplasms - in situ)				0
27	Cancer (neoplasms - malignant)	C00-D48	C00-C97		12
28	Cancer (neoplasms - unknown behaviour)	C00-D48	D37-D48		1
29	Candidal intertrigo	A00-B99	B35-B49		2
30	Candidal vulvovaginitis	A00-B99	B35-B49		1
31	Cardiac enlargement	I00-I99	I30-I52	I51	1
32	Cardiac pacemaker	Z00-Z99	Z80-Z99	Z95	2
33	Carpal tunnel syndrome				0
34	Cellulitis	L00-L99	L00-L08	L03	4
35	Cerebral atrophy				0
36	Cerebrovascular disease	I00-I99	I60-I69		5
37	Cervical myelopathy & cord compression				0
38	Chest infection				0
39	Cholesterol	E00-E90	E70-E90	E78	8
40	Chondrocalcinosis				0

41	Cirrhosis of liver	K00-K93	K70-K77		1
42	Coeliac disease	K99-K93	K90-K93		1
43	Congenital malformations				0
44	Constipation	K00-K93	K59	K59.0	5
45	COPD	J00-J99	J40-J47	J44	8
46	Coronary artery disease				0
47	Cryptogenic fibrosing alveolities				0
48	Cystitis				0
49	Degeneration of lumbar spine				0
50	Dementia	F00-F99	F00-F09		27
51	Depression	F00-F99	F30-F39		9
52	Depression (recurrent)				0
53	Dermatitis	L00-L99	L20-L30		2
54	Diabetes Mellitus (Type 1)	E00-E99	E10-E-14	E10	2
55	Diabetes Mellitus (Type 2)	E00-E90	E10-E14	E11	106
56	Diplegia/Hemiplegia				0
57	Diverticular disease	K00-K93	K55-K63	K57	7
58	Duodenal ulcer				0
59	DVT	I00-I99	I80-I89		5
60	Ear problems	H60-H95			6
61	Eczema	L00-L99	L20-L30	L20	4
62	Electrolyte disorders (eg sodium, potassium)	E00-E90	E70-E90	E87	1
63	Emphysema	J00-J99	J40-J47	J43	1
64	Endocrine disorders (other)	E00-E90	E20-E35		2
65	Epilepsy	G00-G99	G40-G47	G40	2
66	Excessive salivation				0
67	Factor VIII inhibtor activity				0
68	Fractures	S00-T98			4
69	Gall bladder, biliary tract and pancreas	K00-K93	K80-K87		1
70	Gastric haemorrhage	K00-K93	K90-K93		1
71	Gastric ulcer				0
72	Gastritis and duodenitis				0
73	Gastro-oesophageal reflux	K00-K93	K20-K31	K21	2
74	Giant cell arteritis	M00-M99	M30-M36		2
75	Gout	M00-M99	M05-M14	M10	5
76	Haematemesis				0
77	Haematoma				0
78	Haematuria (recurrent & persistent)	N00-N99	N00-N08		1
79	Haemopericardium				0
80	Haemorrhoids	I00-I99	I80-I89	I84	1
81	Hay fever	J00-J99	J30-J39	J30	2
82	Heart block				0

83	Heart defect (electrical)				0
84	Heart failure	I00-I99	I30-I52	I50	15
85	Hemiplegia				0
86	Hernia	K00-K93	K40-K46		1
87	Herpes zoster				0
88	Hiatus hernia	K00-K93	K40-K46		6
89	Hydrocele				0
90	Hydrocephalus				0
91	Hyperplasia of prostate	N00-N99	N40-N51	N40	5
92	Hypertension	I00-I99	I10-I15	I10	41
93	Hypertensive heart disease	I00-I99	I10-I15	I11	5
94	Hyperthyroidism	E00-E90	E00-E07		4
95	Hypopituitarism				0
96	Hypotension	I00-I99	I95-I99	I95	1
97	Hypothyroidism	E00-E90	E00-E07		22
98	Indigestion (dyspepsia)	K00-K93	K20-K31	K30	3
99	Inflammatory arthritis	M00-M99	M00-M25		1
100	Injury and poisoning				0
101	Insomnia	G00-G99	G40-G47	G47	1
102	Intentional self harm				0
103	Interstitial lung disease				0
104	Intestinal obstruction				0
105	Intracerebral haemorrhage				0
106	Intracranial haemmorrhage				0
107	Irritable bladder				0
108	Irritable bowel				0
109	Ischaemic colitis				0
110	Ischaemic heart diseases (IHD)	I00-I99	I20-I25		13
111	Joint pain				0
112	Kyphosis				0
113	Lacerations				0
114	Learning difficulties	F00-F99			1
115	Lewy body dementia				0
116	Lymphoedema (chronic)				0
117	Metabolic disorders	E00-E90	E70-E90		1
118	Microalbuminuria	R00-R99			1
119	Migraine	G00-G99	G40-G47	G43	1
120	Mild cognitive disorder				0
121	Mood (affective) disorders	F00-F99	F30-F39		2
122	MRSA	L00-L99			1
123	Multiple sclerosis				0
124	Muscle contracture	M00-M99	M60-M63		1
125	Myocardial infarction	I00-I99	I20-I25	I21	16
126	Nail disorders				0

127	Nasal polyp	J00-J99	J30-J39	J33	1
128	Neuralgia				0
129	Neutropenia				0
130	Non-compliance				0
131	Not specified				1
132	Nutritional deficiencies	E00-E90	E50-E64		3
133	Obesity	E00-E90	E64-E68		4
134	Oesophagitis	K00-K93	K20-K31	K20	2
135	Oral thrush	A00-B99	B35-B49		2
136	Organic amnesiac syndrome				0
137	Osteoarthritis	M00-M99	M15-M19	M15	26
138	Osteoporosis	M00-M99	M80-M85		6
139	Overactive bladder	N00-N99	N30-N39		1
140	Paget's disease of bone	M00-M99	M80-M94		1
141	Parkinson's disease	G00-G99	G20-G26		5
142	Patulous oesophagus				0
143	Pericardial effusion				0
144	Peripheral vascular disease	I00-I99	I70-I79	I73	5
145	Personality disorder				0
146	Phemphigoid				0
147	Phimosis				0
148	Phlebitis				0
149	Pleural effusion				0
150	Pleural plaque				0
151	Pneumonia				0
152	Polycythaemia				0
153	Polymyalgia rheumatica	M00-M99	M30-M36		5
154	Psychosis				0
155	Pulmonary heart disease	I00-I99	I26-I28		1
156	Rectal bleeding				0
157	Rectal prolapse				0
158	Recurrent UTIs	N00-N99	N30-N39		1
159	Renal failure	N00-N99	N17-N19	N18	33
160	Respiratory failure				0
161	Rheumatoid arthritis	M00-M99	M05-M14		1
162	Schizophrenia	F00-F99			2
163	Scoliosis				0
164	Seizures	G00-G99	G40-G47		1
165	Sepsis				0
166	Shy drager syndromes				0
167	Skin conditions	L00-L99			8
168	Solar keratosis				0
169	Spinal stenonsis				0
170	Spinocerebellar disease	G00-G99	G10-G14		1
171	Spondylosis	M00-M99	M40-M54	M45-M49	1

172	Stroke	I00-I99	I60-I69		24
173	Sudek's atrophy	M00-M99	M80-M94		1
174	Symptoms and signs not elsewhere classified	R0-R99			31
175	Tachycardia				0
176	Transient cerebral ischaemic attack	G00-G99	G40-G47	G45	13
177	Tricuspid regurgitation				0
178	Ulcerative colitis	K00-K93	K50-K52		2
179	Upper respiratory traction infection				0
180	Urinary incontinence	R00-R99			7
181	Urinary reflux				0
182	Urinary tract infection	N00-N99	N30-N39		1
183	Urosepsis				0
184	Uterine prolapse				0
185	Vaginal prolapse				0
186	Varicose veins	I00-I99	I80-I89	I83	4
187	Vascular dementia	F00-F99	F00-F09	F01	11
188	Vasomotor rhinitis				0
189	Venous insufficiency	I00-I99	I80-I89		1
190	Vision impairment/eye conditions	H00-H59			45
191	Missing				980
